# Study on the vasodilatory activity of lotus leaf extract and its representative substance nuciferine on thoracic aorta in rats

**DOI:** 10.3389/fphar.2022.946445

**Published:** 2022-10-05

**Authors:** Hao Deng, Qian Xu, Xiao-Tong Sang, Xing Huang, Li-Li Jin, Fen-Er Chen, Qing-Kun Shen, Zhe-Shan Quan, Li-Hua Cao

**Affiliations:** ^1^ Key Laboratory of Natural Medicines of the Changbai Mountain, Ministry of Education, College of Pharmacy, Yanbian University, Yanji, China; ^2^ Shanghai Engineering Center of Industrial Asymmetric Catalysis for Chiral Drugs, Shanghai, China; ^3^ College of Medical, Yanbian University, Yanji, China

**Keywords:** lotus leaves, nuciferine, vasodilation, thoracic aorta, HUVECs

## Abstract

Lotus (*Nelumbo nucifera*) leaves are widely used for both edible and medicinal applications. For its further utilization, we studied the vasodilatory activity of lotus leaf extract for the first time. In this study, we obtained the extracts using different ratios of water and ethanol, which was followed by polarity-dependent extraction. We found that the CH_2_Cl_2_ layer exhibited better vasodilatory activity (EC_50_ = 1.21 ± 0.10 μg/ml). HPLC and ESI-HRMS analysis of the CH_2_Cl_2_ layer using the standard product as a control revealed that nuciferine (E_max_ = 97.95 ± 0.76%, EC_50_ = 0.36 ± 0.02 μM) was the main component in this layer. Further research revealed that nuciferine exerts a multi-target synergistic effect to promote vasodilation, via the NO signaling pathway, K^+^ channel, Ca^2+^ channel, intracellular Ca^2+^ release, α and β receptors, etc. Nuciferine exhibits good vasodilatory activity, and it exhibits the potential to be utilized as a lead compound.

## Graphical Abstract

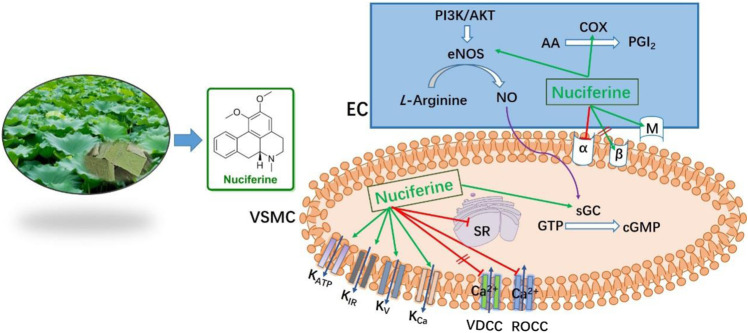

## 1 Introduction

Hypertension is a common chronic disease encountered in clinical practice. Based on the related complications, the mortality and disability rate are at the forefront of human diseases, which causes great harm to human health (Wang et al., 2014; Navaneethabalakrishnan et al., 2020). At present, most blood pressure-lowering drugs in clinical use are chemically synthesized. They exhibit several limitations, such as dependence, high toxicity, side effects, and large fluctuations in blood pressure during blood pressure reduction. Traditional Chinese medicine is known to exhibit less toxicity and side effects can exert an antihypertensive effect through multiple channels and targets, is rich in resources, and has broad development and application prospects. In recent years, the discovery of new active ingredients or lead compounds from natural products for the development of new drugs has gained attention for resolving global concerns and in research.

Lotus leaves are the dried leaves of *Nelumbo nucifera* Geartn*.*, a plant belonging to the Nymphaeaceae family (Liu and Fan, 2014). In recent years, more attention has been paid to the research on elucidating the composition and function of lotus leaves. Its main ingredients include alkaloids, volatile oils, organic acids, and flavonoids (Nguyen and Pham, 1998; Ma et al., 2010; Chen and Qi, 2015; Tungmunnithum et al., 2018; Ye et al., 2018). It has various pharmacological activities, such as regulating fat and weight loss (Ma et al., 2015), anti-inflammatory (Wang M. X. et al., 2015), lowering blood pressure (Ye et al., 2014), anti-oxidation (Chen and Zeng, 2001; Chai et al., 2016; Guo et al., 2020), anti-bacterial (Wang et al., 2007; Yuan et al., 2014), anti-spasmodic (Yang et al., 2018), hemostasis (Chen et al., 2020), and hepatoprotective effects (Guo et al., 2013; Qiu et al., 2020; Wang et al., 2020).

Lotus leaves are widely distributed, rich in resources, and inexpensive. They exert the effect of lowering blood pressure, and their alkaloids have been reported to be involved in relaxation (Wang X. et al., 2015; Yang et al., 2018). However, there has been no systematic research on the active ingredients present in lotus leaves. In this study, we aimed to fill in the knowledge gap on lotus leaf and identify the main components of vasodilatory activity, to ensure more effective use of lotus leaves and provide a basis for its future clinical applications.

## 2 Materials and methods

### 2.1 Materials

Lotus leaf (*Nelumbo nucifera*) was purchased from Hunan Kang Biotech Co. Ltd. and identified taxonomically by Prof. Chang-hao Zhang (College of Pharmacy, Yanbian University). Acetylcholine chloride (Ach), Phenylephrine HCl (PhE), Modulators were purchased from Sigma Chemical Co (St. Louis, MO, United States).

### 2.2 Apparatus

Mass spectrometry (MS) was performed on LTQ Orbitrap XL (Thermo Scientific, United States) in electrospray ionization mode. High-performance liquid chromatography (HPLC) was undertaken on an Ultimate 3,000 (ThermoScientific) system.

### 2.3 Extraction procedures

The purchased lotus leaves were ground into powder. This 10 g of powder was added to a single-necked round-bottomed flask and then 300 ml of various solvents (H_2_O; 25% Ethanol; 50% Ethanol; 75% Ethanol; Ethanol) was added to hot melt at 70°C for 6 h. Extracts were passed through a sand-core filter funnel and evaporated under reduced pressure until dry using a rotary evaporator. All dried extracts were weighed and stored at −20°C until use. The yield was calculated as % yield = (weight of dry extract/initial weight of dry sample) × 100.

### 2.4 Animals and tissues

Animals were strictly handled in accordance with the National Institutes of Health Guidelines for the Care and Use of Laboratory Animals (NIH Publication No. 85–23, revised 1996) and the study was approved by the Institutional Animal Care and Utilization Committee of Yanbian University. Male Sprague-Dawley (SD) rats weighing 250–300 g were used for all experiments, random grouping, 6-8 animals per group. For example, in the intact endothelium experiment, animals were randomly divided into 7 groups with 8 animals in each group (control group, +endo group, Prop group, Atrop group, Indo group, L-NAME group, ODQ group); the experimental results are as follows Figures 6–9. Other animal grouping information was detailed in the Supplementary Information.

SD rats were killed by cervical dislocation. A 3–4 mm long annular segment of the aorta was carefully removed and transferred promptly into ice-cold Krebs solution (NaCl 118.0 mM, KCl 4.7 mm, MgSO_4_ 1.1 mm, KH_2_PO_4_ 1.2 mm, CaCl_2_ 1.5 mm, NaHCO_3_ 25 mm, and glucose 10.0 mm in water, pH 7.4). In some aortic rings, the endothelium was mechanically removed by gently rubbing the surface of the ring back and forth with a plastic tube. Endothelial integrity or functional removal of the constricted blood vessels was confirmed by the presence of relaxation response (over 80%) or its absence (less than 10%) following the treatment with PhE (1 μM) and Ach (1 μM), respectively.

### 2.5 Isometric vascular tone record

The aortic rings were continuously suspended in a tissue bath containing Krebs solution (pH 7.4) and bubbling 95% O_2_–5% CO_2_ at 37°C by inserting 2 stainless steel wires into the lumen of the tube. After an equilibration period of 60 min, under a basal tension of 1 g, the force-displacement sensor (JH-2, Institute of Space Medical Engineering, Beijing, China), connected to the biological laboratory system (Model BL-420S, Cheng-du TME Technology Co., Ltd., Chengdu, China), was used to record changes in isometric tension.

### 2.6 Measurement of the activity of extracts

The contraction of aortic rings was achieved by PhE (1 μM). When the contraction reached a plateau, extracts were added cumulatively (100 μg/ml) to rings with intact endothelium. The relaxation effect was calculated as the percentage of the contraction in response to PhE.

### 2.7 Different solvent extraction and activity detection

The best solvent extract for vasodilation was extracted with PE, CH_2_Cl_2_, EA and *n*-butanol in sequence according to polarity.

### 2.8 HPLC

TSKgel ODS-100V column (C_18_, 3 μm particle size, 4.6 mm I.D × 15 cm) was employed to separate components in CH_2_Cl_2_ extracts. The mobile phase was 0.1% trifluoroacetic acid in acetonitrile (HPLC grade) (A) and water (B) gradient elution (10:90–95:5). The run time was 35 min, the flow rate was 1.0 ml/min, and the detection wavelength was 273 nm. We diluted extract with acetonitrile to give a concentration of 5 mg/ml (Standard nuciferine, 0.5 mg/ml, 2 μL), which was passed through a 0.45-μm filter. The injection volume was 10 μL.

### 2.9 Vascular reactivity assessment

#### 2.9.1 The effect of nuciferine on rat thoracic aorta

To observe the direct effect of nuciferine on the isolated rat aorta, the cumulative concentrations of nuciferine were applied when the tension of thoracic aorta strips was stable, and the vehicle was applied in the control group (Hu et al., 2018).

#### 2.9.2 Vasorelaxant effect of nuciferine on the rat thoracic aorta pre-contracted with PhE

To check the vasorelaxation of nuciferine on the aortic strips with intact or denuded endothelium, after the strips were contracted with PhE (1 μM), a cumulative concentration of nuciferine was added successively into the bath and the changes in the tension were recorded. The percentage of tension change (diastolic ratio) after using nuciferine was calculated as (tension by PhE − tension by nuciferine)/tension by PhE × 100%.

#### 2.9.3 Examination of α_1_-receptor activities

To evaluate whether nuciferine acts as an α_1_-receptor blockade, endothelium-denuded rings were incubated with the nuciferine (0.3, 0.6,1.2 and 2.4 μM) for 20 min before exposing them to PhE (0.1 nM–10 μM), which was added cumulatively. The obtained results are shown as percentages of contraction, and a comparison was done between the results obtained in the absence (control) and the presence of the nuciferine (Cao et al., 2020).

#### 2.9.4 Examination of β and M receptor activities

To assess whether the vasorelaxation induced by nuciferine is associated with β and M receptors, the aortic rings were pre-treated with 1 μM of propranolol or 1 μM of atropine for 20 min before the addition of 1 μM of PhE. The nuciferine (0.03–2.4 μM) was then added cumulatively.

#### 2.9.5 Effects of PGI_2_ on nuciferine-induced relaxation

To explore the possible participation of endothelium-derived prostacyclin (PGI_2_) pathways, the endothelium-intact strips were firstly incubated with 10 μM of Indo, then contracted by PhE (1 μM) and finally a cumulative concentration of nuciferine (0.03–2.4 μM) was added to observe its vasorelaxation (Yang et al., 2020).

#### 2.9.6 Effects of eNOS/sGC/cGMP signaling pathways

To explore the possible participation of endothelium-derived nitric oxide (NO) pathways, the endothelium-intact strips were firstly incubated with *L*-NAME (100 μM) or ODQ (10 μM) or, then, contracted by PhE (1 μM), and finally a cumulative concentration of nuciferine (0.03–2.4 μM) was added to observe its vasorelaxation PhE (1 μM) was precontracted to endothelium-denuded aortic ring and treated with drugs; SNP was used as a positive control. After the experiment, liquid nitrogen quickly freezes the vascular ring. The experimental operations were performed in the presence of the non-specific phosphodiesterase inhibitor IBMX, 10 μM. The frozen aorta was ground in a stainless steel mortar, and the operation was carried out in the presence of liquid nitrogen. Weigh the tissue and add 0.1 M HCl (w/v: 1:10) to homogenize and centrifuge at 1500 g for 15 min, take the supernatant. cGMP level is determined by enzyme immunoassay using acetylation procedure and direct cyclic GMP ELISA kit ADI-900–014 (Kubacka et al., 2018).

#### 2.9.7 Examination of K^+^ channel effect

In order to determine whether nuciferine-induced relaxation was related to the activation of K^+^ channels, we selected four types of K^+^ channel blockers to inhibit the different K^+^ channels, BaCl_2_ (100 μM) for K_IR_, 4-AP (1 mm) for K_V_, TEA (1 mm) for K_Ca_, and Gli (100 μM) for K_ATP_.

#### 2.9.8 Observation of intracellular calcium release

Endothelium-denuded rat thoracic aorta strips were incubated in Ca^2+^ free KBS solution, and then PhE (1 μM) was added to produce the first transient contraction (T_1_). Subsequently, the strips were rinsed with KBS solution 4 times to supplement the intracellular Ca^2+^ loss and with Ca^2+^ free KBS solution for 2 times in succession. Followed that, nuciferine (0.1, 0.3, 0.6 μM) was added and incubated for 10 min and PhE (1 μM) was added again to produce the second transient vasocontraction (T_2_). T_2_/T_1_ × 100% was calculated as contraction (Qu et al., 2015; Hu et al., 2018; Kim et al., 2019).

#### 2.9.9 Observation of extracellular calcium influx

Endothelium-denuded rat thoracic aorta strips were also employed and incubated in Ca^2+^-free KBS. Then 1 μM PhE or 60 mm KCl was added to produce a basic contraction, and 0.01–10 mM of CaCl_2_ were added in sequence to obtain a concentration–response curve. In order to examine the relation of nuciferine-induced vasorelaxation to the contractions by PhE or KCl, nuciferine was pre-incubated for 10 min after adding PhE or KCl. The contraction produced by highest concentration of CaCl_2_ (10 mm) was taken as 100%, and then, based on which, the inhibitory rate of nuciferine could be calculated (Qu et al., 2015; Hu et al., 2018; Kim et al., 2019).

### 2.10 Cell culture

HUVECs were cultured at 37°C on gelatin-coated plates in the basal nutrient media DMEM supplemented with 10% fetal bovine serum, 100 U/ml penicillin, 100 µg/ml streptomycin.

### 2.11 Cell viability assay

Cell viability was determined by a standard MTT (3-(4,5-dimethyl-2-thiazolyl)-2,5.

-diphenyl-2-H-tetrazolium bromide) assay. Briefly, HUVECs were seeded in a 96-well plate at 1 cells/well × 10^4^ cells/well (200 μL). 1, 3, 10, 30 or 100 μM nuciferine treatment for 24 h. MTT solution was added to each well. After 4 h incubation, 150 µL of DMSO was added to dissolve the formazan crystals. Cell viability was reflected by absorbance, which was measured at 492 nm using a microplate reader after 10 min of shaking. Cell viability was expressed as a percentage of the control value. Each experiment was performed in quintuplicate using three independent cultures.

### 2.12 Western blot Analysis

HUVECs were purchased from lonza (Switzerland). HUVECs were pre-incubated with nuciferine, then exposured to TNF-α (10 ng/ml) (Cao et al., 2020). Total cellular proteins were extracted, followed by protein concentration determination using a BCA kit. Followed by protein denaturation, electrophoresis, transferred to a membrane; the membrane was then blocked with a 5% non-fat skim milk solution for 1–2 h. Then incubated with primary and secondary antibodies. Tris-buffered saline and Tween 20 (TBST) was used to wash the membranes for 5 min, which was repeated three times. Densitometry analysis of the protein bands was performed using the ImageJ software.

### 2.13 Statistical analysis

GraphPad Prism 5 software was used for the statistical analysis. All values were expressed as mean ± SD of three independent experiments. When the data were normally distributed, they were analysed by unpaired two-tailed Student’s t-tests and multiple groups were analysed by one-way analysis of variance (ANOVA). A *p* value <0.05 was considered significant.

## 3 Results

### 3.1 Yield of various solvent extracts

**FIGURE 1:**
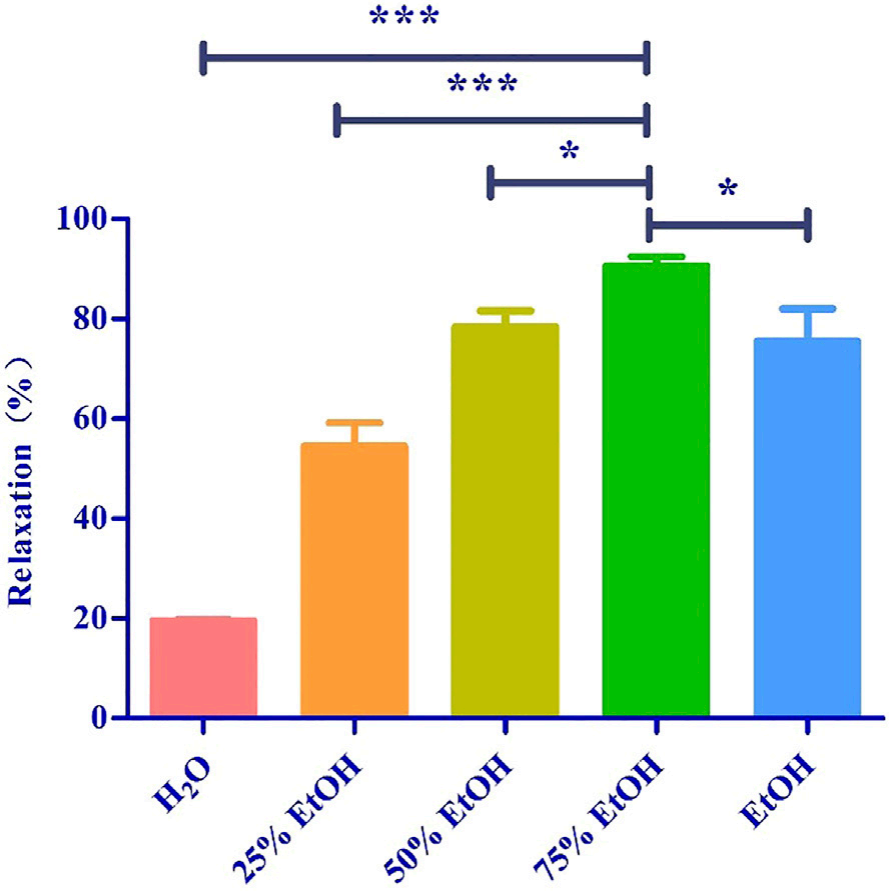
Relaxtion of Lotus leaf with different extraction solvents. The data are expressed as the means ± SD of 3 rats (*n* = 3). ^(*^ mean *p* < 0.05; ^***^ mean *p* < 0.001 vs. 75% Ethanol group).

We investigated the yield of lotus leaf extracts in different solvents. The yield of the H_2_O, 25% ethanol, 50% ethanol, 75% ethanol, and ethanol extracts ranged from 8.94 ± 0.96 to 22.38 ± 1.64% (Table 1). The vasodilatory activity of the H_2_O, 25% ethanol, 50% ethanol, 75% ethanol, and ethanol extracts ranged from 19.65 ± 0.19 to 90.63 ± 1.82% (Table 1 and Figure 1). Based on the yield and vasodilatory activity, 75% ethanol was selected as the most suitable extraction solution.

**TABLE 1:** Extraction yields, and relaxation of Lotus leaf with different extraction solvents.

Extracting solvents	Yields (%, w/w)	Relaxation (10 μg/ml, %)
H_2_O	20.53 ± 0.97	19.65 ± 0.19***
25% Ethanol	19.64 ± 1.74	54.50 ± 4.72***
50% Ethanol	21.21 ± 1.61	78.40 ± 3.25*
75% Ethanol	22.38 ± 1.64	90.63 ± 1.82
Ethanol	8.94 ± 0.96	75.52 ± 6.55*

*Mean *p* < 0.05.

***Mean *p* < 0.001 compared with 75% Ethanol group.

Yield was calculated as % yield = (weight of dry extract/initial weight of dry sample).

### 3.2 Different solvent extractions

**FIGURE 2:**
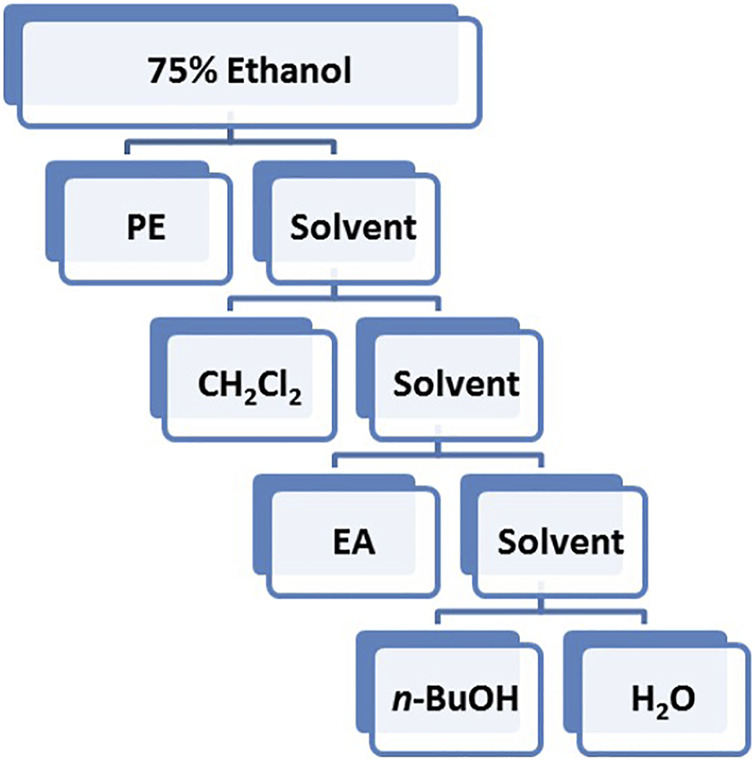
Flow chart of different solvent extraction.

**FIGURE 3:**
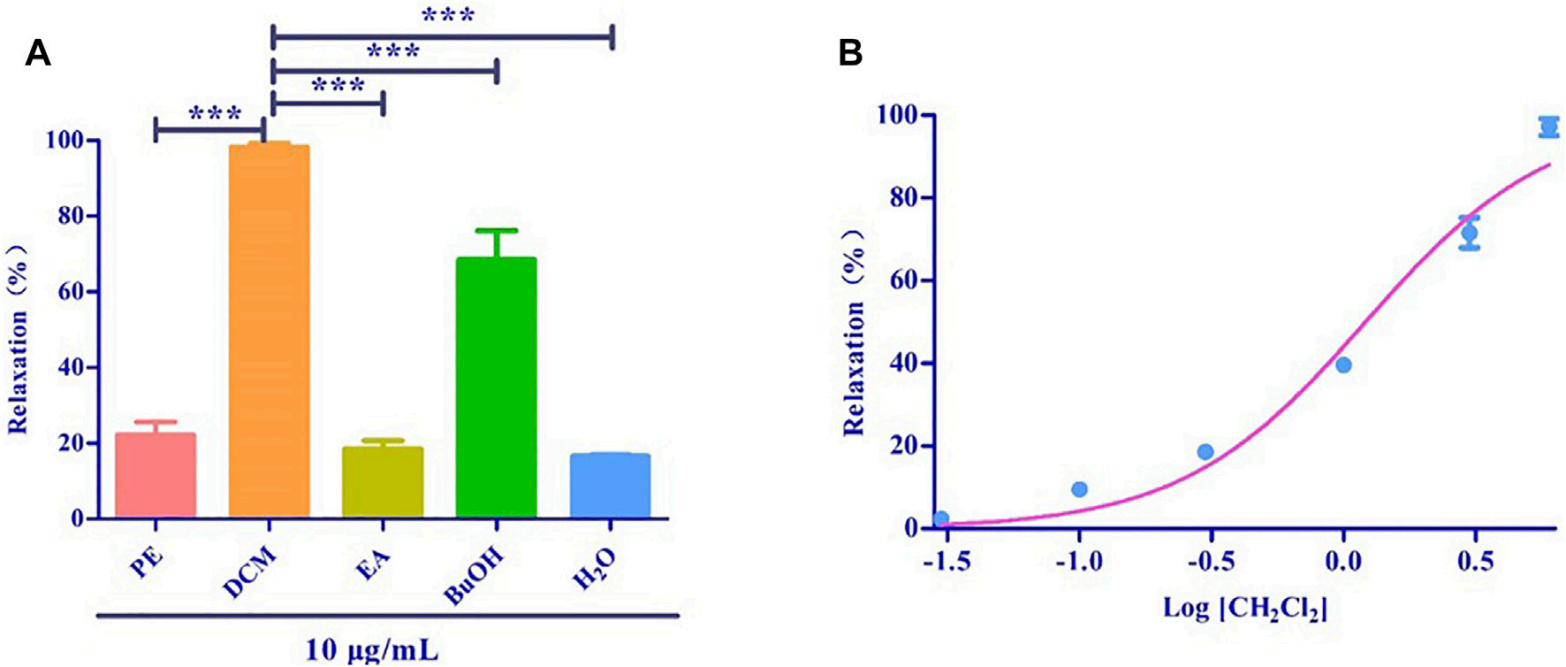
**(A)** Relaxation of different solvent extraction. **(B)** The cumulative concentration-response curve of the effect of CH_2_Cl_2_ (0.03 μg/ml to 6 μg/ml) on isolated rat aortic rings pre-contracted with PhE (1 μM). ^(***^ mean *p* < 0.001 vs. CH_2_Cl_2_ group).

Based on the polarity, the extract obtained using 75% ethanol was further extracted using different solvents (Figure 2). The CH_2_Cl_2_ layer exhibited the best vasodilatory activity (98.22 ± 1.01%) (Table 2 and Figure 3). The vasodilatory activity was concentration-dependent (EC_50_ = 1.21 ± 0.10 µg/ml) (Table 3).

**TABLE 2:** Extraction yields, and relaxation of different solvent extraction.

Extracting solvents	Yields (%, w/w)	Relaxation (10 μg/ml, %)	EC_50_ μg/mL
PE	11.03 ± 1.41	22.20 ± 3.44***	Na
CH_2_Cl_2_	11.79 ± 2.12	98.22 ± 1.01	1.21 ± 0.10
EA	9.60 ± 0.64	18.44 ± 2.27***	Na
*n*-BuOH	26.40 ± 1.36	68.43 ± 7.76***	Na
H_2_O	35.14 ± 1.13	16.55 ± 0.44***	Na

***Mean *p* < 0.001 compared with 75% Ethanol group.

**TABLE 3:** Modulators used for the vasorelaxative effect.

Modulator	Abbreviation	Inhibitory
Atropine	Atrop	Muscarinic receptor (M receptor)
Propranolol	Prop	β-Adrenergic receptor (β receptor)
Indomethacin	Indo	Cyclooxygenase (COX)
*N* ^*G*^-nitro-*L*-arginine methylester	*L-*NAME	nitric oxide synthase (NOS)
1*H*-[1,2,4]-oxadia-zolo-[4,3-a]-quinoxalin-1-one	ODQ	soluble guanylate cyclase (sGC)
Tetraethylammonium	TEA	Ca^2+^-activated K^+^ (K_Ca_) channels
Glibenclamide	Gli	ATP-sensitive K^+^ (K_ATP_) channels
4-Aminopyridine	4-AP	Voltage-dependent K^+^ (K_V_) channels
Barium chloride	BaCl_2_	Inward rectifier K^+^ (K_IR_)channels

### 3.3 HPLC and ESI-HRMS analyses

**FIGURE 4:**
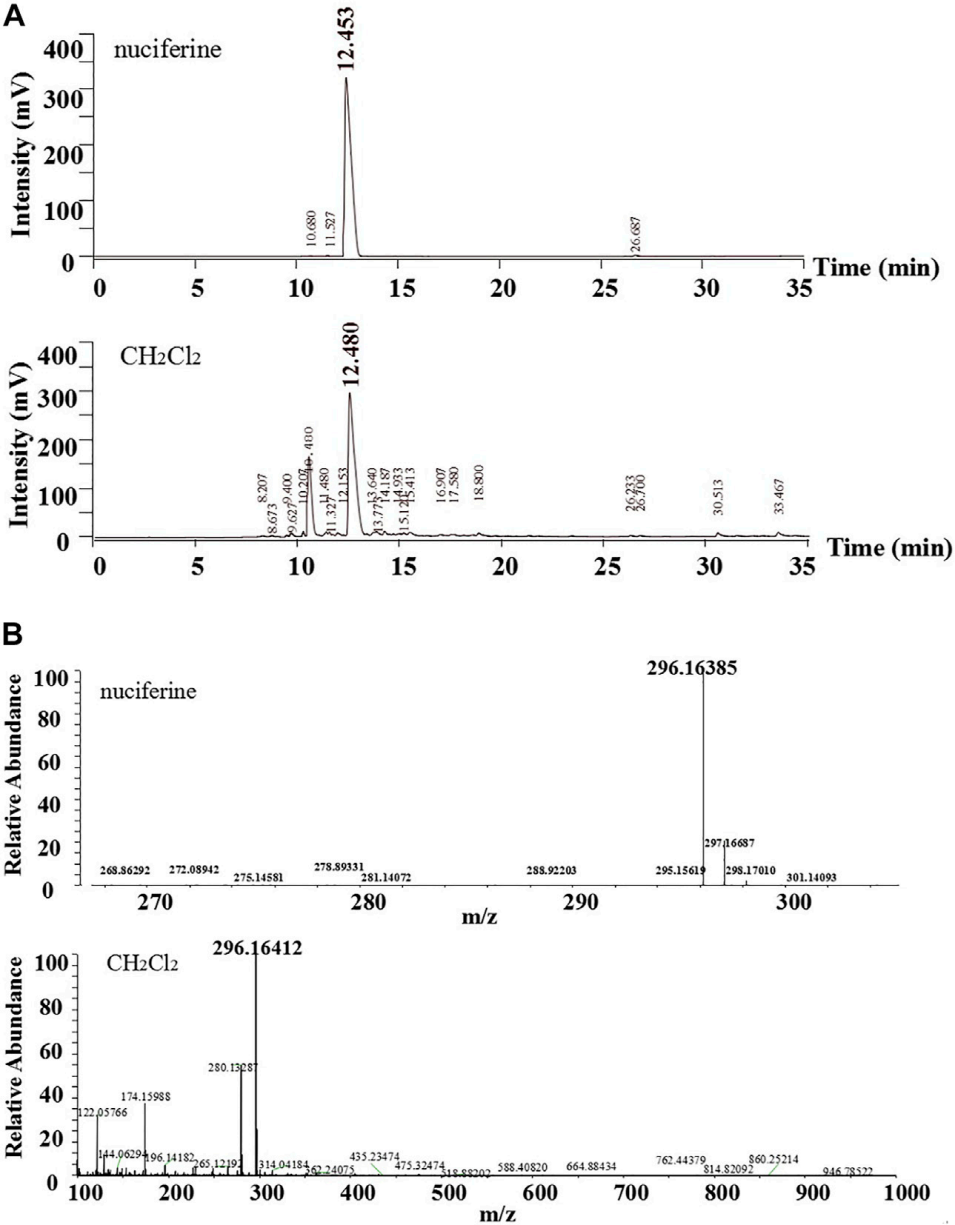
Comparative analysis of nuciferine standard substance and CH_2_Cl_2_ layer **(A)**HPLC; **(B)**ESI-HRMS.

The standard nuciferine product peak appeared at **12.453** min. The component in the CH_2_Cl_2_ layer exhibited a peak at 12.480 min (Figure 4A). The peak shape and time were basically the same. Calculated ESI-HRMS value for nuciferine ([M + H] ^+^) was 296.16451, and it was found to be 296.16385. The found ESI-HRMS value of the component in the CH_2_Cl_2_ layer ([M + H] ^+^) was 296.16412 (Figure 4B).

### 3.4 Vascular reactivity assessment

#### 3.4.1 Effect of nuciferine on rat thoracic aorta

**FIGURE 5:**
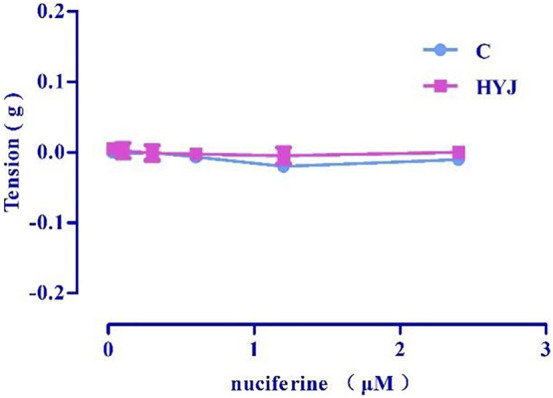
The effect of concentration-dependent addition of nuciferine on blood vessels. The data are expressed as the means ± SD of 6–8 rats (*n* = 6–8).

Accumulation of nuciferine (0.03–2.4 μM) had no obvious effects on basal tension in rat thoracic aortic rings when compared to the vehicle control group (Figure 5).

#### 3.4.2 Nuciferine relaxes the pre-contracted PhE in rat thoracic aorta

**FIGURE 6:**
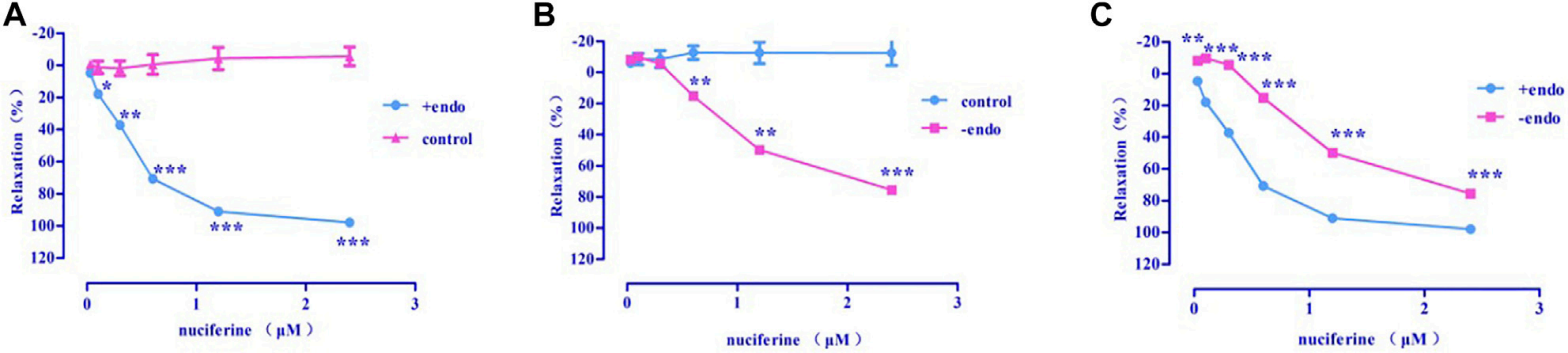
The vasodilation effect of nuciferine **(A)** Intact endothelium (+endo); **(B)**. Endothelium-denuded (-endo); **(C)** ±endo. The data are expressed as the means ± SD. of 6–8 rats (n = 6–8). (*p < 0.05; **p < 0.01 and ***p < 0.001 vs. control group).

Nuciferine exerted concentration-dependent vasorelaxant effect on PhE induced contraction in both endothelium-intact (E_max_ = 97.95 ± 0.76%, EC_50_ = 0.36 ± 0.02 μM) (Figure 6A) and endothelium-denuded (E_max_ = 75.42 ± 1.83%, EC_50_ = 1.30 ± 0.03 μM) (Figure 6B) arteries. Therefore, the vasodilatory activity of nuciferine is thought to be related to the endothelium (Figure 6C).

#### 3.4.3 Examination of α-, β-, and M-receptor activities

##### 3.4.3.1 Examination of α-receptor activity

**FIGURE 7:**
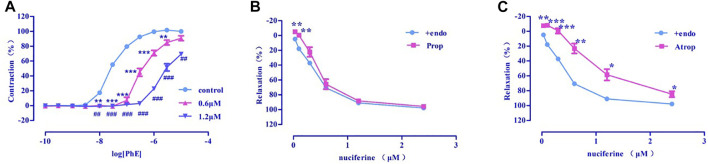
**(A)** α-receptor. **(B)** β receptor. **(C)** M receptor. The data are expressed as the means ± SD of 6–8 rats (*n* = 6–8). (*p < 0.05, **p < 0.01 and ***p < 0.001 vs. control group; ^#^ *p* < 0.05, ^##^ *p* < 0.01 and ^###^ *p* < 0.001 vs. control group).

Pre-incubation with various concentrations of nuciferine (0.6, 1.2 μM) significantly inhibited the concentration-response contraction induced by PhE (0.1 nm–10 μM), and suppressed its maximal contraction (E_max_) to 90.73 ± 3.26% and 69.39 ± 1.59%, respectively (vs. control group E_max_ = 100%) (Figure 7A). The vasodilatory activity of nuciferine is thought to be related to the α-receptor activity.

##### 3.4.3.2 Examination of β- and M-receptor activities

There was no significant difference between propranolol pre-incubated vascular ring (E_max_ = 95.66 ± 1.30%, EC_50_ = 0.48 ± 0.02 μM) (Figure 7B) and the control group (E_max_ = 97.95 ± 0.76%, EC_50_ = 0.36 ± 0.02 μM). The vasorelaxation induced by nuciferine is thought to be associated with M-receptor activity (E_max_ = 84.67 ± 4.06%, EC_50_ = 1.05 ± 0.03 μM) (Figure 7C).

#### 3.4.4 Effects of PGI_2_ on nuciferine-induced relaxation

**FIGURE 8:**
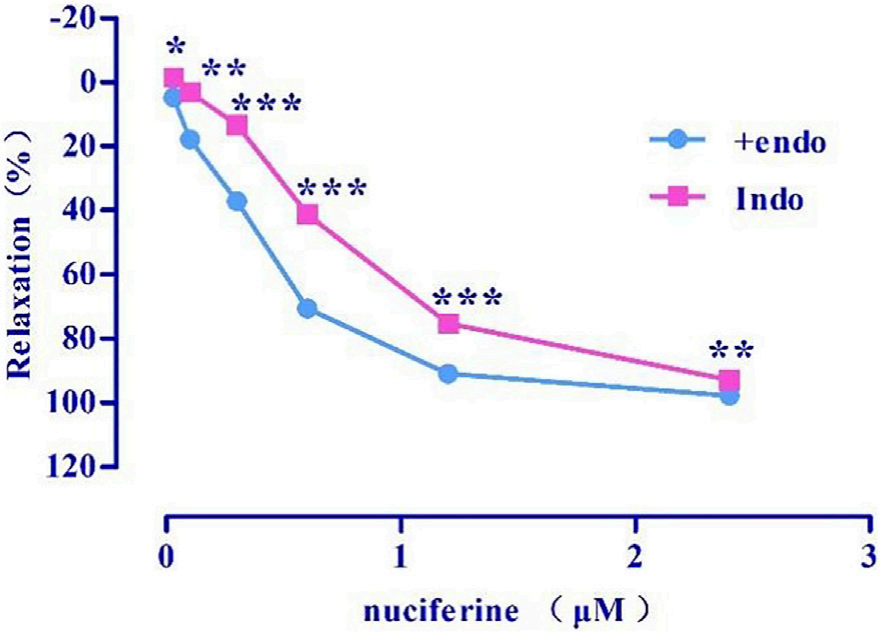
Effect of Indo on nuciferine induced vasorelaxation. The data are expressed as the means ± SD of 6–8 rats (*n* = 6–8). (*p < 0.05, **p < 0.01 and ***p < 0.001 vs. control group).

Indo (E_max_ = 92.98 ± 0.31%, EC_50_ = 0.71 ± 0.01 μM) (Figure 8) pretreatment weakened the relaxing effect of nuciferine. Therefore, the vasodilatory effect of nuciferine may be related to PGI_2_.

#### 3.4.5 Effect of eNOS/sGC/cGMP on nuciferine-induced relaxation

**FIGURE 9:**
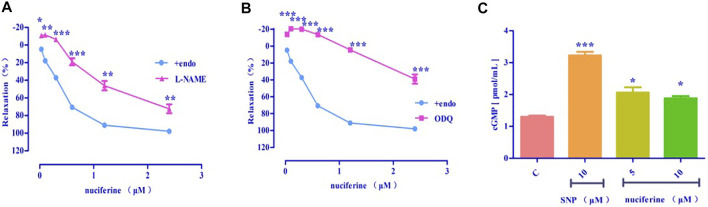
Effect of eNOS/sGC/cGMP on nuciferine-induced relaxation **(A)** *L*-NAME blocking effect; **(B)** ODQ blocking effect; **(C)** Detection of cGMP content. The data are expressed as the means ± SD of 6–8 rats (*n* = 6–8). (*p < 0.05, **p < 0.01 and ***p < 0.001 vs. control group).

After pretreatment with eNOS inhibitor *L*-NAME (E_max_ = 72.43 ± 5.00%, EC_50_ = 1.36 ± 0.04 μM) (Figure 9A) and sGC inhibitor ODQ (E_max_ = 39.01 ± 5.44%) (Figure 9B), the vasodilatory effect of nuciferine was found to be significantly reduced. The inhibitory effect of ODQ is stronger than that of *L*-NAME, and it is suspected that nuciferine can directly act on smooth muscle cells. The endothelium was pretreated with lotus leaf alkaline extract to remove the blood vessels, and then, the cGMP content was assessed. The results revealed that there were significant differences between the nuciferine group (5 μM, 2.06 ± 0.24 pmol/ml; 10 μM, 1.88 ± 0.10 pmol/ml) and the control group (1.30 ± 0.05 pmol/ml). The cGMP content of the positive control SNP was 3.23 ± 0.16 pmol/ml (10 μM) (Figure 9C). The vasodilatory effect of nuciferine is thought to be related to the eNOS/sGC/cGMP signaling pathway. The cGMP results also proved that lotus leaf can directly activate sGC in smooth muscle cells to increase the production of cGMP.

#### 3.4.6 Examination of K^+^ channel effect

**FIGURE 10:**
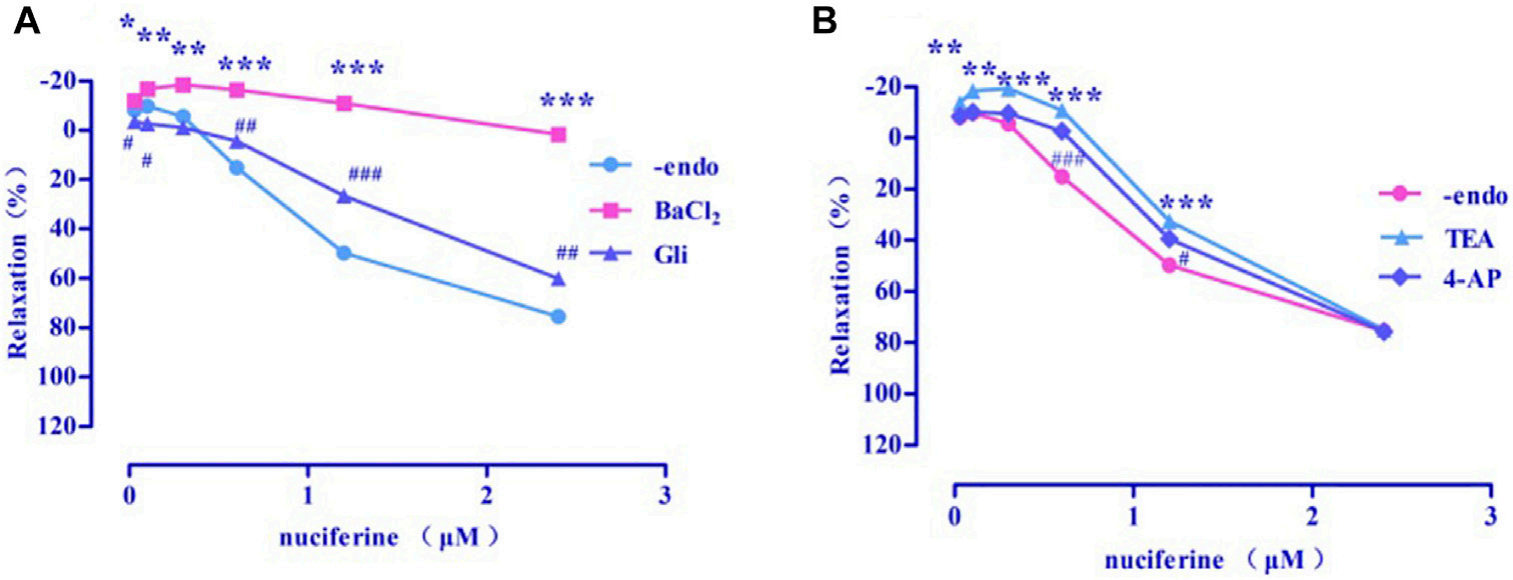
Effects of K^+^ channel blockers on nuciferine induced vasorelaxation. The data are expressed as the means ± SD of 6–8 rats (*n* = 6–8). (*p < 0.05, **p < 0.01 and ***p < 0.001 vs. control group; ^#^ *p* < 0.05, ^##^ *p* < 0.01 and ^###^ *p* < 0.001 vs. control group).

Four types of selective K^+^ channel blockers, including BaCl_2_ (E_max_ = 1.73 ± 2.48%), Gli (E_max_ = 60.23 ± 2.30%, EC_50_ = 1.97 ± 0.01 μM), TEA (E_max_ = 74.93 ± 0.47%, EC_50_ = 1.63 ± 0.04 μM), and 4-AP (E_max_ = 75.80 ± 1.60%, EC_50_ = 1.50 ± 0.02 μM) were added into the incubation solution to obtain endothelium-denuded strips (E_max_ = 75.42 ± 1.83%, EC_50_ = 1.30 ± 0.03 μM) separately. The results revealed that all these K^+^ channel blockers affected the nuciferine-induced relaxation (Figures 10A,B), especially BaCl_2_.

#### 3.4.7 Effect of sarcoplasmic reticulum calcium release

**FIGURE 11:**
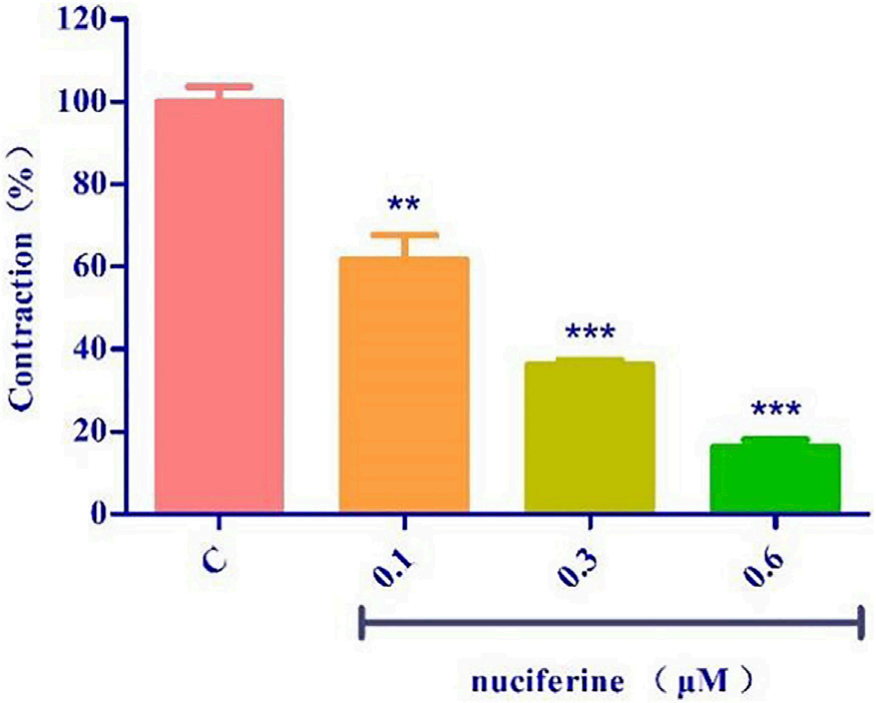
Effects of SR calcium release on nuciferine induced vasorelaxation. The data are expressed as the means ± SD of 6–8 rats (*n* = 6–8). (**p < 0.01 and ***p < 0.001 vs. control group).

The endothelium-denuded strips were incubated in a Ca^2+^-free solution and contracted by PhE, which promoted calcium release from the SR. The pre-treatment with nuciferine (0.1, 0.3, and 0.6 μM) significantly affected the PE-mediated vasocontraction (Figure 11). This result suggested that nuciferine interferes with SR Ca^2+^ release for its vasodilation upon considering the control group as 100%.

#### 3.4.8 Effect of extracellular Ca^2+^ influx on vasorelaxation by nuciferine

##### 3.4.8.1 Examination of ROCCs effect

**FIGURE 12:**
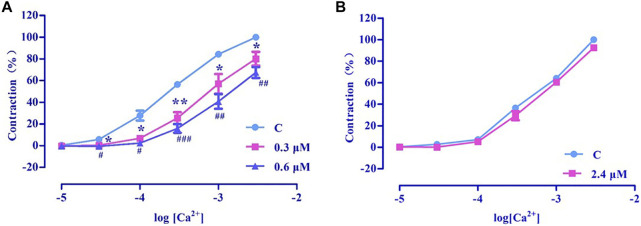
Effects of extracellular Ca^2+^ influx on nuciferine induced vasorelaxation. The data are expressed as the means ± SD of 6–8 rats (*n* = 6–8). (*p < 0.05 and **p < 0.01 vs. control group; ^#^ *p* < 0.05, ^##^ *p* < 0.01 and ^###^ *p* < 0.001 vs. control group).

PhE promotes Ca^2+^ influx via ROCCs. The results revealed that nuciferine (0.3 and 0.6 μM) affected the contraction induced by CaCl_2_ + PhE (Figure 12A), suggesting that the relaxation by nuciferine might be related to the ROCCs.

##### 3.4.8.2 Examination of VDCCs effect

High concentration of KCl promoted Ca^2+^ influx via VDCCs. The results revealed that 2.4 μM nuciferine did not affect the contraction induced by CaCl_2_ + KCl (E_max_ = 91.68 ± 1.01%) (Figure 12B), thereby suggesting that the relaxation by nuciferine might not be related to VDCCs.

### 3.5 Cell viability assay

**FIGURE 13:**
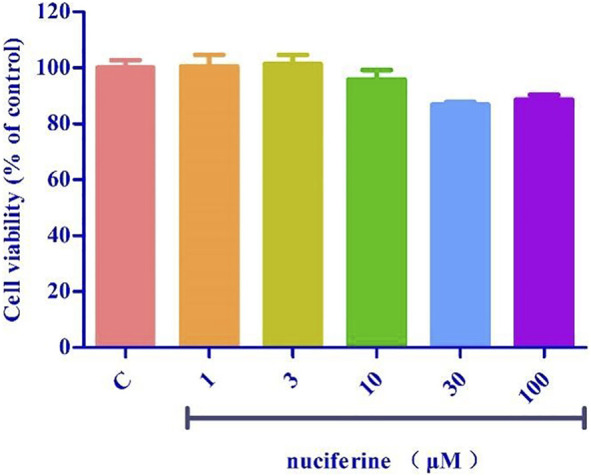
Toxicity test of nuciferine on HUVECs.

Pretreatment with nuciferine (1–100 μM) for 24 h did not result in significant difference between the control group (Figure 13).

### 3.6 Vascular protection effect of nuciferine

**FIGURE 14:**
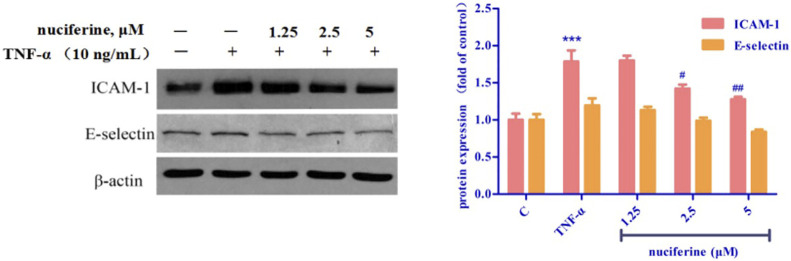
Effect of nuciferine on TNF-α-induced expression of adhesion molecules in HUVECs. (*p < 0.01 and ***p < 0.001 vs. control group).

TNF-α treatment increased the expression of cell adhesion molecules, such as ICAM-1 and E-selectin. Interestingly, pretreatment with nuciferine attenuated TNF-*α*-mediated cell adhesion molecules (Figure 14). Our results revealed that nuciferine may exert a vascular protective effect.

## 4 Discussion

Lotus leaves contain alkaloids, flavonoids, polysaccharides, and other ingredients (Nguyen and Pham, 1998; Ma et al., 2010; Chen and Qi, 2015; Tungmunnithum et al., 2018; Ye et al., 2018). Different ratios of water and ethanol were used to obtain the extracts, and the 75% ethanol extract was found to exert a strong vasodilator effect. The 75% ethanol extract was further subjected to polarity-dependent extraction, and it was found that the CH_2_Cl_2_ layer exhibited the highest vasodilatory activity. Therefore, the component analysis of the CH_2_Cl_2_ layer was carried out, and the components that accounted for about half of the entire peak area in the liquid phase analysis were separated by preparative liquid phase. The chromatographic characterization and the comparative analysis of liquid phase/mass spectrometry confirmed that the substance was nuciferine, which was consistent with previous research results that most of the alkaloids were dissolved in the dichloromethane layer (Wang et al., 2008) and nuciferine was the most abundant alkaloid in lotus leaf (Ma et al., 2010). Before this, some scholars have studied the relaxation mechanism of nuciferine on mesenteric arteries (Wang X. et al., 2015) and trachea (Yang et al., 2018). With this, we believe that nuciferine is the main component present in lotus leaves with vasodilator activity. Therefore, the thoracic aorta activity of nuciferine was further studied. In this study, we aimed to provide a basis for future studies on the vasodilatory activity of lotus leaf extracts.

As shown in Figure 6, nuciferine was found to directly act on the rat thoracic aorta without pretreatment, and there was no significant effect. The vasodilatory effect of nuciferine is more related to the endothelium. The vascular endothelium produces vasodilatory factors, such as NO and PGI_2_ (Yam et al., 2016). When eNOS is activated, *L*-arginine is converted into NO in vascular endothelial cells. NO penetrates the endothelium and enters the smooth muscle cells, and then, activates the soluble guanylate cyclase, thereby increasing the intracellular levels of cGMP, which is synthesized from guanosine triphosphate (Qu et al., 2015; Kubacka et al., 2018). The thoracic aortic vasorelaxant effect of nuciferine was also blocked in the *L*-NAME and ODQ experiments, which is consistent with the trend of nuciferine to relax rat mesenteric arteries (Wang X. et al., 2015). But in the thoracic aorta experiment, the blocking effect of ODQ was more obvious. Based on the above phenomenon, nuciferine may directly activate sGCs in smooth muscle cells to promote cGMP production. The nitric oxide donor SNP was selected as a positive control, and nuciferine directly treated smooth muscle cells. The detection of cGMP content in vessels found that nuciferine could directly act on smooth muscle cells, then relate vessels. In conclusion, the vasodilatory effect of nuciferine may be related to the NO signaling pathway.

As another endogenous vasodilator prostacyclin (PGI_2_) synthesized and released by endothelial cells, both PGI_2_ and NO act directly on smooth muscle cells. However, the mechanism of action of the two is slightly different. PGI_2_ binds to smooth muscle cell membrane receptors and promotes the production of cAMP to relax vessels (Moncada et al., 1976; Gomberg-Maitland and Olschewski, 2008). Arachidonic acid (AA) is converted to PGI_2_ by COX (Yam et al., 2016). In the present study, Indo was found to partially inhibit the relaxation effect of nuciferine. This is different from previous findings that the relaxation of mesenteric arteries by nuciferine is independent of PGI_2_ (Wang X. et al., 2015). This suggested that the relaxation mechanism of nuciferine in the thoracic aorta and mesenteric arteries was different, and proved that the relaxation of the thoracic aorta by nuciferine may be related to the PGI_2_ signaling pathway.

Ions play an important role in maintaining blood vessel homeostasis (Qu et al., 2015; Hu et al., 2018). We found that four different types of potassium channels are related to the vasodilatory effect of nuciferine, especially the inwardly rectifying potassium channels. In most cases, the contraction of the vascular smooth muscle is related to an increase in [Ca^2+^] ions through the influx of extracellular Ca^2+^ and the release of intracellular Ca^2+^. The influx of extracellular calcium is mainly through the VDCCs and ROCCs located in the cell membrane. The KCl-induced contraction is mainly caused by the depolarization and opening of the VDCCs membrane, which drives the influx of extracellular Ca^2+^. PhE leads to the contractions in response to the extracellular Ca^2+^ influx upon direct activation of ROCCs. We found that the vasodilatory effect of nuciferine is related to the ROCCs pathway of intracellular calcium release and extracellular calcium influx (Kim et al., 2015; Lee et al., 2015; Kim et al., 2019).

Nuciferine relaxes the thoracic aorta through endothelium-dependent and -independent mechanisms. Therefore, nuciferine may act directly on endothelial cells and smooth muscle cells. α- and β-receptors are widely distributed in vascular smooth muscle cells, and muscarinic (M) receptors are distributed in the vascular endothelium. Stimulation of α-receptor results in vasoconstriction, and M- and β-adrenergic receptors are involved in vasodilation (Chiu et al., 2004; Yam et al., 2016). β-adrenergic receptors were not involved in the vasodilatory effect of nuciferine, which was consistent with mesenteric arteries findings (Wang X. et al., 2015). The present study demonstrates that the vasodilatory effects of nuciferine are associated with α- and M-receptors.

A variety of cardiovascular diseases such as hypertension, atherosclerosis and heart failure are often accompanied by endothelial dysfunction (Vanhoutte, 1996). TNF-α is reported to trigger the interaction between monocytes and vascular endothelial cells, enhance the expression of adhesion molecules, such as VCAM-1, ICAM-1, and E-selectin, and finally induce endothelial dysfunction (Yao et al., 2015; Zhou et al., 2017). Nuciferine did not show cytotoxicity at the concentration of 0–100 μM, and the results of western blot experiments showed that nuciferine inhibits the production of vascular adhesion factors in a concentration-dependent manner and protects vessels.

In summary, nuciferine was the main substance with a vasodilatory effect in the lotus leaf extract. The vasodilatory effects of nuciferine were mediated by multiple pathways, multiple-targets and had both endothelium-dependent and -independent mechanisms. Nuciferine mainly mediated the NO/cGMP signaling pathway, activated the K_IR_ channel, and inhibited ROCCs and SR Ca^2+^ release to relax thoracic aorta. Notably, Nuciferine has low cytotoxicity and endothelial protective effect, which was worth further development and research. This study provides a new research direction for the development and application of lotus leaf extract.

## Data Availability

The original contributions presented in the study are included in the article/Supplementary Material, further inquiries can be directed to the corresponding authors.

## Abbreviations

Ach, acetylcholine chloride; PhE, phenylephrine HCl; MS, mass spectrometry; HPLC, high-performance liquid chromatography; PE, petroleum ether; CH_2_Cl_2_, dichloromethane; EA, ethyl acetate; PGI_2_, prostacyclin; NO, nitric oxide; SNP, sodium nitroprusside; IBMX, 3-isobutyl-1-methylxanthine; cGMP, guanosine 3′,5′-cyclic monophosphate; AA, arachidonic acid; COX, cyclooxygenase; VDCCs, voltage-dependent Ca^2+^ channels; ROCCs, receptor-operated Ca^2+^ channels; TNF-α, tumor necrosis factor-α; Modulators (Table 3).
