# Supplementary material for: Study on the vasodilatory activity of lotus leaf extract and its representative substance nuciferine on thoracic aorta in rats
Source: Front Pharmacol. 2022 Oct 5;13:946445. doi: 10.3389/fphar.2022.946445 (PMC9581309; doi:10.3389/fphar.2022.946445)
Supplement: Supplementary file 1 [file DataSheet1.docx]

**Supplementary information**

The specific grouping of this study:

1. In the preliminary screening experiment of drug extracts, animals were randomly divided into 5 groups with 3 animals in each group. The experimental results were shown in Fig. **1**.

2. In the experiment of extracting drugs with different solvents, the animals were randomly divided into 7 groups with 8 animals in each group; **3A** shows the data of 5 groups; **3B** shows the data of the other two groups. The experimental results were shown in Fig. **3**.

3. The direct effect of lotus leaf alkaloid on blood vessels is divided into 2 groups, with 8 animals in each group. The experimental results were shown in Fig **5.**

4. In the endothelium-denuded experiment, animals were randomly divided into 6 groups with 8 animals in each group (control group, -endo group, BaCl_2_ group, Gli group, TEA group). The experimental results were shown in Fig **6B**, Fig **10**.

5. In the α-receptor experiment, the animals were randomly divided into 3 groups with 8 animals in each group (control group, 0.6 μM nuciferine group, 1.2 μM nuciferine group). The experimental results were shown in Fig **7A**.

6. In the cGMP content determination experiment, the animals were randomly divided into 4 groups with 8 animals in each group (control group, 10 μM SNP group, 5 μM nuciferine group, 10 μM nuciferine group). The experimental results are shown in Fig **9C**.

7. In the intracellular Ca^2+^ release experiment, animals were randomly divided into 4 groups, control group, 0.1 μM nuciferine group, 0.3 μM nuciferine group, and 0.6 μM nuciferine. The experimental results were shown in Fig **11**.

8. In the ROCC experiment, the animals were randomly divided into 3 groups, control group, 0.3 μM nuciferine group, and 0.6 μM nuciferine group. In the VDCC experiment, the animals were randomly divided into two groups, control group and 2.4 μM nuciferine group. The experimental results were shown in Fig **12**.
